# Using a quitline plus low-cost nicotine replacement therapy to help disadvantaged smokers to quit

**DOI:** 10.1136/tc.2008.026492

**Published:** 2009-01-08

**Authors:** C L Miller, V Sedivy

**Affiliations:** 1 The Cancer Council South Australia, Adelaide, Australia; 2 School of Population Health and Clinical Practice, University of Adelaide, South Australia, Australia

## Abstract

**Objectives:**

To trial an intervention in a real-life setting to motivate low-income smokers to try to quit. The intervention under trial was the addition of subsidised nicotine replacement therapy (NRT) to a standard population quitline service.

**Design:**

Participants were low-income smokers, recruited “cold” via either a letter in the mail or a flyer inserted in a local newspaper. The intervention group received the usual service of multisession counselling from the quitline plus access to heavily subsidised NRT. A comparison group received the usual quitline service only. Participants were followed up at 3, 6, and 12 months. Trial participants were also compared with a sample of general callers to the quitline.

**Results:**

The offer of subsidised NRT recruited more than twice as many low-income smokers than the offer of the cessation service alone (intervention group n = 1000; comparison group n = 377). 63% were first-time callers to the quitline. Intervention respondents showed higher levels of nicotine dependence than comparison group respondents. Comparisons of quitting data were confounded by the differences in the respondents at baseline. 73.5% of smokers in the intervention group tried to quit compared to 61.0% in the comparison group. Unadjusted quit rates were higher in the intervention group than in the comparison group at 3 months and 6 months but not at 12 months.

**Conclusions:**

Disadvantaged smokers were easily engaged to call the quitline, particularly when offered subsidised NRT. Disadvantaged smokers using the quitline, with and without subsidised NRT, achieved cessation outcomes comparable to other studies of “mainstream” smokers.

Smoking rates in lower socioeconomic groups continue to be a major concern to health authorities.^1^ While the effectiveness of quitlines in providing cessation support to smokers is well established,^2^ the relevance and/or accessibility of such services for disadvantaged groups is unknown.

The provision of subsidised, or free, nicotine replacement therapy (NRT) is proposed as a potential strategy to assist disadvantaged smokers to quit. In general populations, smokers assert that price is a major impediment to accessing NRT.^3^ Therefore, the cost of NRT in low-income smokers may be a significant impediment to smoking cessation. Primary economic principles indicate that when the price of ordinary goods drops demand increases. Thus, lowering the price of NRT could potentially lead to an increase in volume sold; may improve access and use of NRT in lower socioeconomic groups; and may, ultimately, lead to improved rates of smoking cessation at the population level.

The question for policymakers in tobacco control is whether subsidies for the purchase of NRT for lower socioeconomic groups would lead to increased access and use of NRT, and ultimately decreased rates of smoking. NRT is an efficacious cessation aid^4^ on its own and when combined with behavioural support.^5^ Some tobacco control programmes have deliberately added subsidised NRT to behavioural support to try to improve cessation rates. The provision of low-cost or no-cost NRT is an integral component of quitline services offered in many North American states^6^ as well as in New Zealand.^7^

Several studies indicate that the provision of no-cost or low-cost NRT with quitline services has led to an increase in general demand for quitline services.^8^^–^11 However, it is not clear to what extent the provision of low-cost NRT prompts individuals from lower socioeconomic groups to contact a quitline service. Callers to the Oregon quitline were not made aware of free NRT until after they had contacted the service.^12^ Following the promotion of free NRT, the Ohio quitline observed an increase in demand but a significant decline in the proportion of callers from lower socioeconomic groups was noted.^13^

In this paper, we report on the results of an observational study of a pilot trial of subsidised NRT, delivered via a quitline service. The trial aimed to target smokers in lower socioeconomic groups. In addition to providing demographic data, patterns of utilisation of quitline services, and NRT, smoking behaviours are described.

## METHODS

### Purposive sampling frame: targeting lower socioeconomic groups

Recruitment was conducted during October through to December 2005.

A random sample of potential individual participants was selected from the two lowest socioeconomic quintiles of the South Australian electoral roll. In Australia, voting is compulsory and the Australian Electoral Commission estimates that 93.6% of eligible South Australian adults were enrolled to vote in March 2006.^14^

### Letters of invitation

Letters of offer to participate in a “free quit smoking service …” were sent to individuals’ home addresses, as listed on the electoral role. Letters differed in that half of the letters included an invitation relevant to receiving the standard quitline service, whereas half of the letters also offered a subsidy for the use of nicotine patches, gum or other NRT product.

### Inclusion criteria

In order to participate, subjects were required to be 18 years of age or older; current smokers; smoking more than 10 cigarettes per day; willing to receive telephone support for quitting; and willing to participate in three follow-up interviews by phone.

To ensure that the subjects captured were of a lower socioeconomic status, they were required to be in possession of an Australian Government concession card. This card is only provided to individuals who are in receipt of low to very low incomes (compared to the national average).

### Exclusion criteria

The NRT group was screened for contraindications. In Australia in 2005, NRT stated contraindicators were: recently had a heart attack or stroke; breast feeding or pregnant; or received advice by physician not to use NRT. Participants were free to withdraw their participation at any time.

### Pilot trial incorporating low-cost NRT

The comparison group comprised those participants who responded to the invitation to participate in the quitline’s standard service whereas the NRT group comprised those participants who responded to the invitation to receive the standard service plus the subsidised NRT. These participants at study entry were mailed vouchers in packs of 10 (that is, equivalent to one week’s worth of NRT) for redemption of NRT products at a subsidised rate—that is, 75% off the usual recommended retail price.

Both groups had equal access to the standard quitline programme that incorporated multiple-session counselling. Quitline counsellors delivered standard quitline counselling. The number and length of sessions is determined by the caller and their needs, so long as the counsellors believe that good behavioural progress is being made. Multi-session counselling would not go on for more than 12 weeks.

### Amendments to sampling protocol

#### Self-selected sample

Limited responses (that is, n = 111 in response to the standard service invitation and n = 249 to the NRT subsidy invitation) led the investigators to incorporate additional sampling methods. Letters of invitation were inserted as an A4 flyer into free community newspapers delivered to households, identified by the Australian Bureau of Statistics^15^ as low-income areas. In total, 150 000 standard service invitations and 150 000 NRT invitations were distributed. Standard service and NRT invitation inserts were alternately inserted into the newspapers.

#### Regular quitline sample

In addition, a further sample was derived by selecting all regular callers to the quitline during October 2005 to December 2005. The regular quitline sample was not necessarily disadvantaged and did not receive any special invitation or offer to call in. Not all of the demographic data of regular quitline callers, collected during the study, were collected routinely; where data were collected, they are given here.

### Measures

Participants were interviewed using a semi-structured interview on three occasions. Demographic data (gender, age, education and cultural background) along with information on smoking behaviour (number of cigarettes smoked, quit attempts) and previous use of quitline services were collected. Unless otherwise specified, quit rates refer to one-day point prevalence quit rates. Period quit rates are defined as having quit for an entire period (for example, between the 3-month and 6-month follow-up surveys), with no relapse. Other self-report data relevant to NRT use, and perceptions of cessation services using NRT were collected.

### Procedure

When the initial contact was made with the quitline counsellors, individuals were screened for eligibility according to the selection and exclusion criteria. It was known that callers were ringing in response to the invitation because they were given a special line to call in on.

Initial data were collected by quitline staff; however, follow-up data were collected at 3 months, 6 months and 12 months by independent research unit staff. During each follow-up, researchers attempted to reach each participant by telephone up to six times on different days and at different times of day, including weekends and evenings. Because the 3-month follow-up questionnaire included questions about satisfaction with the service they received, including receipt of NRT, researchers could not be blinded to which arm participants were in during this follow-up.

#### Methods used to maintain retention in study

Several attempts were made to follow up participants by telephone at 3 months, 6 months and 12 months. People who could not be contacted at one follow-up, despite several attempts, were still included in the sample to be recontacted at subsequent follow-ups.

### Statistical analyses

Analyses were undertaken using SPSS v15. Tests for between-group differences were done using χ^2^ tests for nominal data and t tests for continuous data.

## RESULTS

### Response rates

In total, 1000 participants were recruited to the NRT group and 377 to the comparison group. Combining both methods of recruitment, the response rates were NRT group—0.67% (1000/150 000) and comparison group 0.25% (377/150 000). The response rate in the NRT group was significantly higher than in the comparison group (χ^2^ = 283; p<0.001).

Of these 1377 participants, 1192 (87%) were successfully reached for the 3-month follow-up interview consisting of 863 NRT group participants and 329 comparison group participants; at 6 months we reached 1137 people (83%; 832 NRT group; 305 comparison group); and at 12 months we reached 929 (67%; 672 NRT group; 257 comparison group). No significant differences were observed in the response rates between the NRT group and the comparison group at any follow-up.

### Characteristics of the study population at baseline

Overall, 63% of participants were first-time callers to the quitline, with no difference evident between the two groups. As shown in table 1, the groups did not differ in any of the demographic characteristics though those in the NRT group were significantly less likely to have made a quit attempt in the past year, were heavier smokers and were more likely to smoke within 30 minutes of waking.

**Table 1:** Characteristics of the study population at baseline

Sociodemographics	Intervention (n = 1000)	Comparison (n = 377)	p Value
Gender (female)	65.3%	62.1%	NS
Age (mean, years)	48.3	49.7	NS
Indigenous (Australian Aboriginal or Torres Strait Islander)	1.1%	2.4%	NS
**Education**			NS
Left school at 15 years or less	25.7%	25.7%	
Left school after age 15	38.0%	35.3%	
Still studying	1.9%	2.4%	
Certificate/diploma	19.8%	18.8%	
Trade/apprenticeship	9.4%	12.7%	
Bachelor degree or higher	4.8%	4.8%	
Unknown/missing	0.4%	0.3%	
**Smoking behaviour at baseline**			
Years smoked (mean (SD))	30.8 (14.0)	31.7 (14.4)	NS
Cigarettes per day (mean (SD))	24.9 (9.8)	23.5 (9.4)	<0.05
First cigarette of day (within 30 minutes of waking)	87.3%	82.4%	<0.05
**Previous quitting experience at baseline**			
Ever tried to quit (% yes)	95.0%	95.0%	NS
Made serious attempt to quit in past year (% yes)	49.6%	56.0%	<0.05
Ever contacted quitline (% yes)	37.9%	35.5%	NS

### Use of NRT and multisession counselling

When interviewed at 3 months, 98.5% of the NRT group reported receiving vouchers for NRT; 1.5% of participants reported they did not receive vouchers but the majority reported that vouchers arrived soon after contacting the quitline (98.5%).

According to the quitline records, 10 170 vouchers were distributed, and pharmacy records indicate that 3741 vouchers were redeemed, yielding an overall redemption rate of 36.8%. Among those who received vouchers, 80.9% (n = 686) reported using at least one of them. This figure corresponds closely to data from the quitline and pharmacy records (79.2%). The mean number of vouchers redeemed among the NRT group was 4.9 (self-report; or 5.1, quitline and pharmacy records).

#### Reasons for non-redemption

NRT group participants who did not redeem any vouchers (n = 162) or all of their vouchers (n = 571) were asked about reasons for non-redemption. Of those who redeemed at least one voucher, but not all, 29.9% reported relapsing before using them all, 23.3% were still in the process of using NRT at the time of the 3-month follow-up and 18.6% reported having quit successfully without needing all the NRT available. Of those who did not redeem any NRT vouchers, 30.9% reported never having made a quit attempt and 25.3% reported attempting to quit without using NRT. The vast majority of those who did not use any or all of the NRT vouchers reported keeping them (94.0%) rather than discarding them (2.3%), giving them away (1.1%) or returning them (0.4%).

A total of 51 participants (7.4% of those who received the vouchers) reported having trouble with the voucher redemption process; the main obstacle reported was trouble getting transport to the pharmacy (n = 46).

The comparison group was not prevented or discouraged from using NRT as the choice to use NRT is consistent with recommendations offered by the standard quitline service. As shown in table 2, the use of NRT was prevalent in both groups, although much higher in the NRT group. Furthermore, the majority of study participants were contacted at least once for a proactive cessation call from quitline staff, with no significant difference evident between study groups. However, participants in the NRT group received more quitline calls than participants in the comparison group.

**Table 2:** Use of nicotine replacement therapy (NRT) and multisession counselling

Self-reported NRT use	Intervention	Comparison	p Value
**Among all participants**	(n = 1000)	(n = 377)	<0.05
Yes	57.9%	22.3%	
No	7.3%	29.4%	
NA, did not try to quit	21.1%	35.5%	
Not reached at 3-month follow-up	13.7%	12.7%	
**Among participants who (tried to) quit**	(n = 652)	(n = 195)	<0.05
Yes	88.8%	43.1%	
No	11.2%	56.9%	
**Duration of use of NRT**	(n = 579)	(n = 84)	
Mean days (SD)	38.8 (26.0)	22.2 (22.0)	<0.05
**Quitline counselling**	(n = 1000)	(n = 377)	
1 or more calls from quitline	94.7%	95.9%	NS
Number of calls from quitline, mean (SD)	6.6 (3.7)	5.8 (3.9)	<0.001

NA, not applicable.

#### Smoking behaviours

Table 3 provides data on average numbers of cigarettes smoked, among those still smoking at each follow-up. The average number of cigarettes smoked per day among both groups who reported smoking at each follow-up remained significantly lower than at baseline. While the average number of cigarettes smoked increased significantly in the NRT group between 3 months and 12 months and 6 months and 12 months, corresponding increases over time in the comparison group were not statistically significant.

**Table 3:** Number of cigarettes smoked per day, among those who were smoking at 3-month and 6-month follow-ups

	Intervention	Comparison	p Value
Average number smoked (baseline) (n_c_ = 232; n_i_ = 466)	24.9 (9.5)	24.4 (9.6)	NS
Average number smoked (3 months) (n_c_ = 232; n_i_ = 466)	14.5*** 10.1)	15.7*** (10.6)	NS
Average number smoked (6 months) (n_c_ = 223; n_i_ = 519)	15.1*** (9.6)	16.4*** (9.7)	NS
Average number smoked (12 months) (n_c_ = 181; n_i_ = 443)	17.6*** (9.6)	16.9*** (9.7)	NS

Values are mean (SD).

***Change between baseline and follow-up is statistically significant at p⩽0.001.

#### Efforts to quit

Table 4 reports on data collected on quitting attempts and outcomes. The data are presented in two forms—responder estimate (that is, using those who were able to be recontacted at the relevant follow-up as a denominator) and a conservative estimate (that is, using the baseline sample as the denominator, which assumes that any respondents not reached for follow-up did not make a quit attempt). Table 4 shows that those in the NRT group were more likely to have made an attempt to quit at some time before the 12-month follow-up.

**Table 4:** Quitting behaviour and outcomes

	Intervention (%)	Comparison (%)	p Value
**Responder estimates**			
Attempted to quit (n_i_ = 918; n_c_ = 345)	83.8	74.8	⩽0.001
Quit at 3 months (n_i_ = 863; n_c_ = 329)	46.0	29.5	⩽0.001
Quit at 6 months (n_i_ = 832; n_c_ = 305)	37.1	26.2	⩽0.001
Quit at 12 months (n_i_ = 672; n_c_ = 257)	33.2	28.0	NS
Period prevalence (sustained quitting from 3-month to 6-month follow-ups) (n_i_ = 861; n_c_ = 329)	20.7	13.1	⩽0.01
Period prevalence (sustained quitting from 3-month to 12-month follow-ups) (n_i_ = 748; n_c_ = 303)	2.7	2.0	NS
**Conservative estimate**s			
Attempted to quit (conservative estimate: n_i_ = 1000; n_c_ = 377)	76.9	68.4	⩽0.001
Quit at 3 months (n_i_ = 1000; n_c_ = 377)	39.7	25.7	⩽0.001
Quit at 6 months (n_i_ = 1000; n_c_ = 377)	30.9	21.2	⩽0.001
Quit at 12 months (n_i_ = 1000; n_c_ = 377)	22.3	19.1	NS
Period prevalence 3–6 months (n_i_ = 1000; n_c_ = 377)	2.0	1.6	NS
Period prevalence 3–12 months (n_i_ = 1000; n_c_ = 377)	2.0	1.6	NS

n_i_, number in sample (that is, denominator) for intervention group; n_c_, number in sample for comparison group.

Statistically higher quit rates were observed among the NRT group at 3-month and 6-month follow-ups but differences observed at the 12-month follow-up were not significant. Period prevalence (that is, continued abstinence from smoking) was calculated using the 3-month follow-up interview as a starting point and the 6-month and 12-month follow-ups as endpoints. The 3-month interview was chosen as the start point to allow for the majority of participants to have received the full complement of quitline services, incorporating the proactive callback service that often entails preparation for quitting.

As evident in table 4, period prevalence fell sharply in both groups after the 6-month assessment, and overall differences between study groups were minimal.

#### Use of resources and 6-month quitting outcomes

Use of resources was examined by study group (NRT group vs comparison group) and by quitting outcome at 6 months. Overall, the NRT group received slightly more callbacks on average than the comparison group (6.3 vs 5.5 calls, p<0.001). Further analyses revealed that while in both groups those who had quit received more proactive callbacks than those who had continued to smoke, there was no difference in the number of callbacks received by those who quit in the NRT group (7.8 callbacks) and those who quit in the comparison group (7.7 callbacks).

Within the NRT group, those who had quit (at 6 months) used a significantly larger number of NRT vouchers than those who did not quit (6.0 compared to 4.2; t = 7.6; df = 625; p<0.001).

#### Regular quitline sample

There were 503 callers to the regular quitline during the study period. Overall, 67.0% of regular quitline callers (337 callers) were from the lowest two socioeconomic quintiles. This compares to 58.6% of all smokers in South Australia (Hickling J, personal communication 13 November 2008). Sixty per cent of regular callers were female and they smoked an average of 23.7 cigarettes per day (SD 12.6). When compared with the sample of routine callers to the South Australian quitline, callers who were recruited using trial methodology and meeting trial criteria used more resources than routine callers. Routine callers received an average of 2.6 (SD 1.9) counselling callbacks, substantially less than the comparison group in the trial.

#### Disadvantaged smokers’ appraisal of cessation services offered

Support for the notion of subsidised NRT was very strong among all trial participants. When participants in both groups were asked about how important they thought offering subsidised NRT would be as a strategy for helping most smokers quit, 98.4% of the NRT and 95.4% of the comparison group thought that it would be somewhat or very helpful for most smokers. Support for government subsidies for discount on NRT was very high with 99.0% (NRT group) and 98.8% (comparison group) endorsing this strategy.

When asked about the telephone counselling and proactive callbacks they received, 95.4% of study participants found the quitline advisers to be very friendly. Overall, 85.9% of study participants found the callback service somewhat or very helpful and 84.2% reported that the number of proactive calls that they received was about right. There was a significant difference observed (χ^2^ = 6.2; df = 2; p<0.04) in the appraisal of the appropriateness of the number of calls received between groups, with the intervention group being more likely to report having received too many calls than the comparison group (11.4% compared to 8.0%) and the comparison group being more likely to report receiving too few (7.4% compared to 4.5% of the intervention group). Overall, the majority of trial participants (87.9%) reported that they did not find the proactive calls to be intrusive or inconvenient.

## DISCUSSION

In this study, it was evident that the provision of subsidised NRT was a significant motivator to contact quitline services. Participation rates among recruits offered NRT were 2.5 times higher than those offered the comparison service. Moreover, the NRT group displayed characteristics at baseline consistent with greater dependence on smoking and this finding suggests that the offer of an NRT subsidy may act as an incentive for more dependent smokers to contact a quitline service.

The pilot trial deliberately targeted individuals from lower socioeconomic groups because of their high smoking rates. The sample of regular quitline callers demonstrated that lower socioeconomic groups are already over-represented among callers to the quitline, suggesting that this demographic group already utilises the regular quitline service. Comparison with the regular quitline callers further reinforces the role of subsidised NRT as an incentive to call. Over the same period, the regular quitline received 337 calls from disadvantaged smokers, the comparison or standard service invitation prompted 377 calls from disadvantaged smokers and the NRT invitation prompted 1000 calls from disadvantaged smokers.

The smoking cessation services delivered (with and without NRT) were very well appraised by participants. Most gave a positive appraisal of the quitline service and its proactive callbacks demonstrating that statewide telephone cessation services are acceptable to disadvantaged smokers.

Those participants purposively selected used more resources—namely, more counselling, than general callers to the quitline. Within the study itself, volume of calls was predicted by cessation outcome rather than study group; those who had quit at 6 months having used more callbacks than those who continued to smoke, irrespective of study group. Counsellors were not blinded to the fact that the callers were in the trial, so it is possible that extra efforts were made with these group. However, the counsellors used in the trial were experienced and they were well briefed to deliver counselling in line with standard practice and protocols.

Together, this demonstrates that a statewide telephone cessation service is flexible enough to be adapted to the needs of disadvantaged smokers, but it can be anticipated that this group of smokers will have higher needs resulting in a more resource intensive intervention. Furthermore, the cessation outcomes achieved by these disadvantaged smokers (in both trial groups) were equivalent to those observed in other prospective studies of studies of general quitline callers (that is, point prevalence of 29% at 12 months).^16^ Hence, disadvantaged smokers are just as capable of quitting as other smokers and the quitline is just as able to assist disadvantaged smokers to quit, albeit with greater intensity of assistance.

Despite attempts to randomise participants to the NRT arm or the standard quitline arm, the two groups differed significantly on baseline smoking behaviours—namely, mean cigarettes per day, time to first cigarette per day and serious attempts at quitting in the past year. The confounding of results compromises the comparison of quitting outcomes. A substantial number of callers in both groups did quit smoking and a further number cut down. What can be shown from this trial is that the NRT subsidy recruited more (and heavier) smokers and resulted in 223 quitters versus 72 for the comparison group.

Previous research has shown that the introduction of free NRT has also been shown to improve 6-month point prevalence quit rates in cohorts of quitline callers, compared with quit rates in cohorts of callers to the same services before the introduction of free NRT.^11^ ^17^ A comparison of callers to the New York State smokers’ quitline who received NRT and a non-random comparison group of callers who did not receive NRT due to mailing errors showed higher cessation rates among NRT recipients.^13^

Caution about the long-term cost-effectiveness of a widespread roll-out of low-cost or no-cost NRT is necessary. Quitlines offering subsidised NRT have experienced increased costs per quitter because of the cost of the NRT itself, but also because of increased use of counselling.^9^ ^12^ A randomised trial on the Oregon quitline found that the additional counselling and NRT costs were offset by increased effectiveness of cessation outcomes.^17^ Because of its extraordinary impact on call volume, favourable comparisons have been drawn between mass media and offering free NRT as a cost-effective mechanism to drive calls to the quitline.^17^

**What this paper adds**

Inequalities in smoking rates between richer and poorer groups are a public health concern. The relevance or adequacy of “mainstream” cessation services for disadvantaged smokers is sometimes questioned. This study demonstrates that although the level of intervention needed was higher, the quitline offered an acceptable, relevant service that assisted a socioeconomically disadvantaged group of smokers to quit. Offering subsidised NRT was a strong incentive for lower-income smokers to call.

In summary, the real potential benefit of offering disadvantaged smokers subsidised NRT through a state quitline, as was shown in this study, is that it appears to act as an incentive (or motivator) to seek assistance with quitting. Once the disadvantaged smokers made the call and accessed the quitline, they found the service appropriate to their needs and were just as able to quit as routine callers to the service. If promoting quit attempts is fundamental to increasing the cessation rate, then offering low-cost NRT helps recruit disadvantaged smokers to an effective cessation service to facilitate those attempts. This study does not make comparisons between the cost of NRT as a quitline recruitment strategy compared with other methods such as mass media advertising. The costliness of NRT needs to be balanced against other proved strategies to promote quitting among disadvantaged smokers. However, it may well be worth investigating the cost-effectiveness of offering low-cost NRT as a complement to mass media advertising to promote quit attempts and to recruit to mainstream cessation services for this disadvantaged group of smokers.
